# Extracranial Carotid Plaque Hemorrhage Is Independently Associated With Poor 3-month Functional Outcome After Acute Ischemic Stroke—A Prospective Cohort Study

**DOI:** 10.3389/fneur.2021.780436

**Published:** 2021-12-14

**Authors:** Fengli Che, Yanfang Liu, Xiping Gong, Anxin Wang, Xiaoyan Bai, Yi Ju, Binbin Sui, Jing Jing, Xiaokun Geng, Xingquan Zhao

**Affiliations:** ^1^Department of Neurology, Beijing Tiantan Hospital, Capital Medical University, Beijing, China; ^2^Department of Neurology, Beijing Luhe Hospital, Capital Medical University, Beijing, China; ^3^Tiantan Neuroimaging Center for Excellence, Beijing Tiantan Hospital, Capital Medical University, Beijing, China; ^4^Department of Neuroradiology, Beijing Tiantan Hospital, Capital Medical University, Beijing, China; ^5^Department of Neurology, China-America Institute of Neuroscience, Beijing, China; ^6^Research Unit of Artificial Intelligence in Cerebrovascular Disease, Chinese Academy of Medical Sciences, Beijing, China

**Keywords:** MRI, atherosclerosis, plaque, prognosis, acute ischemic stroke

## Abstract

**Background and Purpose:** Carotid plaque hemorrhage (IPH) is a critical plaque vulnerable feature. We aim to elucidate the association between symptomatic extracranial carotid atherosclerotic IPH and poor 3-month functional outcome after acute ischemic stroke by high-resolution vessel wall MRI (HRVMRI).

**Methods:** We prospectively studied consecutive patients with a recent stroke or transient ischemic attack (TIA) of carotid atherosclerotic origin. All patients underwent a High-Resolution (HR) VWMRI scan of ipsilateral extracranial carotid within 1 week after admission. The patients recruited were interviewed by telephone after 3 months after stroke onset. The primary outcome was a 3-month functional prognosis of stroke, expressed as a modified Rankin Scale (mRS) score. A poor prognosis was defined as a 3-month modified Rankin Scale (mRS) score ≥ of 3. Univariate analysis was used to analyze the correlation between risk factors and IPH. The relation between IPH and 3-month functional outcome was analyzed by Logistic regression analysis.

**Results:** A total of 156 patients (mean age, 61.18 ± 10.12 years; 108 males) were included in the final analysis. There were significant differences in the age, gender, smoking history, national institutes of health stroke scale (NIHSS) on admission, and diastolic blood pressure (DBP) on admission between the IPH group and the non-IPH group (all *p* < 0.05). During the follow-up, 32 patients (20.5%) had a poor functional outcome. According to the prognosis analysis of poor functional recovery, there was a significant difference between the two groups [36.7 vs. 16.7%; unadjusted odds ratio (OR), 2.32, 95% confidence interval (CI), 1.12–4.81, *p* = 0.024). Even after adjusting for confounding factors [such as age, gender, smoking history, National Institutes of Health Stroke Scale (NIHSS) on admission, DBP on admission, stenosis rate of carotid artery (CA), calcification, loose matrix, lipo-rich necrotic core (LRNC), and statins accepted at 3 months], IPH was still a strong predictor of poor 3-month outcome, and the adjusted OR was 3.66 (95% CI 1.68–7.94, *p* = 0.001).

**Conclusions:** Extracranial carotid IPH is significantly associated with poor 3-month outcome after acute ischemic stroke and can predict the poor 3-month functional prognosis.

## Introduction

Atherosclerosis is the leading cause of ischemic stroke in the Chinese population (1). The Chinese Intracranial Atherosclerosis Study (CICAS) showed that 14% of patients with ischemic stroke had severe extracranial carotid atherosclerosis (> of 50%). Some studies have confirmed that carotid artery atherosclerotic stenosis is significantly associated with the onset and prognosis of acute ischemic stroke (2, 3). However, histopathological studies have demonstrated that the vulnerable features of carotid artery plaque are the underlying risk factors of most ischemic events (4–10). Therefore, identifying the vulnerable plaques based on advanced medical imaging technology and exploring the correlation between the vulnerability of the plaque and stroke recurrence has become research hotspots in recent years. Meanwhile, we still do not know whether the characteristics of carotid artery plaques in acute ischemic stroke are associated with the poor 3-month outcome of patients after stroke onset.

Our study aims to analyze the vulnerable characteristics of carotid plaque by high-resolution vessel wall magnetic resonance imaging (HR VWMRI) and find a relation between IPH and the early poor prognosis of acute ischemic stroke. With that in mind, we might identify the risk factors of poor 3-month outcome of stroke and take more effective individualized prevention strategies in the acute stage of ischemic stroke.

## Methods

### Study Population

All patients were enrolled from a single-center prospective cohort study in Beijing Tiantan Hospital. We aimed to assess the characteristics of carotid atherosclerotic plaques and the relationship between IPH and poor 3-month outcome after stroke onset. All enrolled patients underwent an ultrasonographic screen and a HR VWMRI scan of the symptomatic extracranial carotid artery within 1 week after admission. They were followed up by telephone after 3 months (90 ± 7 days) after stroke onset. According to the latest international guidelines, we gave the enrolled patients standardized medical therapy and secondary prevention of stroke. In addition, the inclusion criteria and exclusion criteria of the study have been mentioned in the Supplementary Material. The Ethics Committee approved the study of Beijing Tiantan Hospital. All study subjects had signed written consent.

### Carotid MR Imaging Protocol

All recruited subjects were scanned using 3.0 T MR scanners (Achieva TX; Philips Healthcare, Best, the Netherlands) with 8-channel head coils. A standardized multisequence protocol included three-dimensional time of flight (3D-TOF), T1-weighted quadruple inversion recovery, T2-weighted multislice double, and magnetization prepared gradient recalled echo (MPRAGE) imaging sequences. We used gadolinium diethylenetriamine pentametric acid (GD-DTPA) as the contrast medium, and the injection dose was 0.1 mmol/kg. We used spectral preservation attenuated inversion recovery (SPAIR) in the black blood technique. The study's imaging parameters of carotid atherosclerotic plaque MRI have also been mentioned in the Supplementary Material.

### MRI Image Analysis

We used a standard workstation (ADW4.4, G.E. Medical System, USA) for imaging analysis. Two experienced senior neuroimaging physicians reviewed all slices of multi-contrast MR vessel wall images. They were both blinded to the diffusion-weighted images (DWI) and clinical information. The plaque was defined as a thickening of the focal wall relative to image slices beneath the T2 and T1-weighted imaging focal wall. We manually outlined the outer wall boundaries and lumen of carotid arteries at each slice. The parameters of plaque morphology and the stenosis ratio of the carotid were measured. According to the international criteria detailed previously, the plaque components such as carotid artery (CA), lipo-rich necrotic core (LRNC), IPH, and fibrous cap rupture (FCR) were detected by MRI (11). MR image quality was divided into a four-point grade (1 = poor; 2 = general; 3 = good; and 4 = excellent), and slices with image quality ≥ 3 were included in the statistical analysis. We would accept consensus interpretation as the final analysis if inconsistent in the two readers' interpretations. We randomly selected twenty patients from the study population every 2 months to test the consistency of inter-reader and intra-reader in measuring carotid plaque morphology and carotid plaque compositions.

### Outcome Assessment

Patients were followed up at 3 months after stroke onset. The primary outcome was 3-month functional outcome, expressed as a modified Rankin Scale (mRS) score 3 months after discharge. A poor functional outcome was defined as mRS score ≥of 3 at 3 months. The secondary outcomes were early stroke progression, early recurrent ischemic stroke, and hemorrhagic transformation at 3 months post-discharge. The recurrence of ischemic stroke was confirmed according to new neurological deficits documented in the medical records. Two experienced stroke neurologists reviewed patients' medical documents to ensure a reliable diagnosis of recurrent ischemic stroke. The early stroke progression was described as an incremental increase in the NIHSS score by ≥2 points in the total score or ≥1 point in motor power within 1 week after admission (excluding hemorrhage transformation of cerebral infarction and symptomatic intracranial hemorrhage), accompanied by the new ischemic signals on brain MRI or computed tomography (CT) (12). Early recurrent ischemic stroke (ERIS) was defined as the occurrence of an ischemic stroke in other independent arterial regions confirmed by clinical symptoms or by CT/MRI imaging techniques (13). Hemorrhagic transformation was defined as the hemorrhage in the infarct area or the corresponding vascular distribution area after acute cerebral infarction (14). Complications during hospitalization were defined as at least one syndrome, namely, urinary infection, pulmonary infection, gastrointestinal bleeding, deep vein thrombosis, other organ dysfunction, and/or acute coronary syndromes within 7 days. Patients or their authorized proxies were interviewed at 3 months by telephone by trained research coordinators. Two trained researchers in Beijing Tian Tan hospital completed all the telephone follow-ups of patients. Trained stroke neurologists assessed the NIHSS. The mRS at the 3-month time point was evaluated by trained and experienced neurologists by a telephone assessment using a standardized structured questionnaire. An experienced neurologist independently confirmed all the followed-up assessments. They were also blinded to imaging information of all patients.

### Statistical Analysis

Continuous variables were described by means [standard deviations (SDs)] or medians [interquartile ranges (IQRs)], and categorical data were expressed as frequency and percentage. To analyze the difference between the IPH group and the non-IPH group, Student's *t*-test or Mann–Whitney *U*-test were used for continuous variables according to a normal distribution, and chi-squared test or Fisher exact test for categorical variables. Univariate and multivariable Cox regression analyses were conducted to study predictors of poor prognosis of stroke by calculating odds ratio (OR) and 95% confidence interval. Statistical analyses were performed using SPSS version 22.0 (IBM, NY, USA). A two-sided *p* < 0.05 was considered statistically significant.

## Results

**Figure 1:**
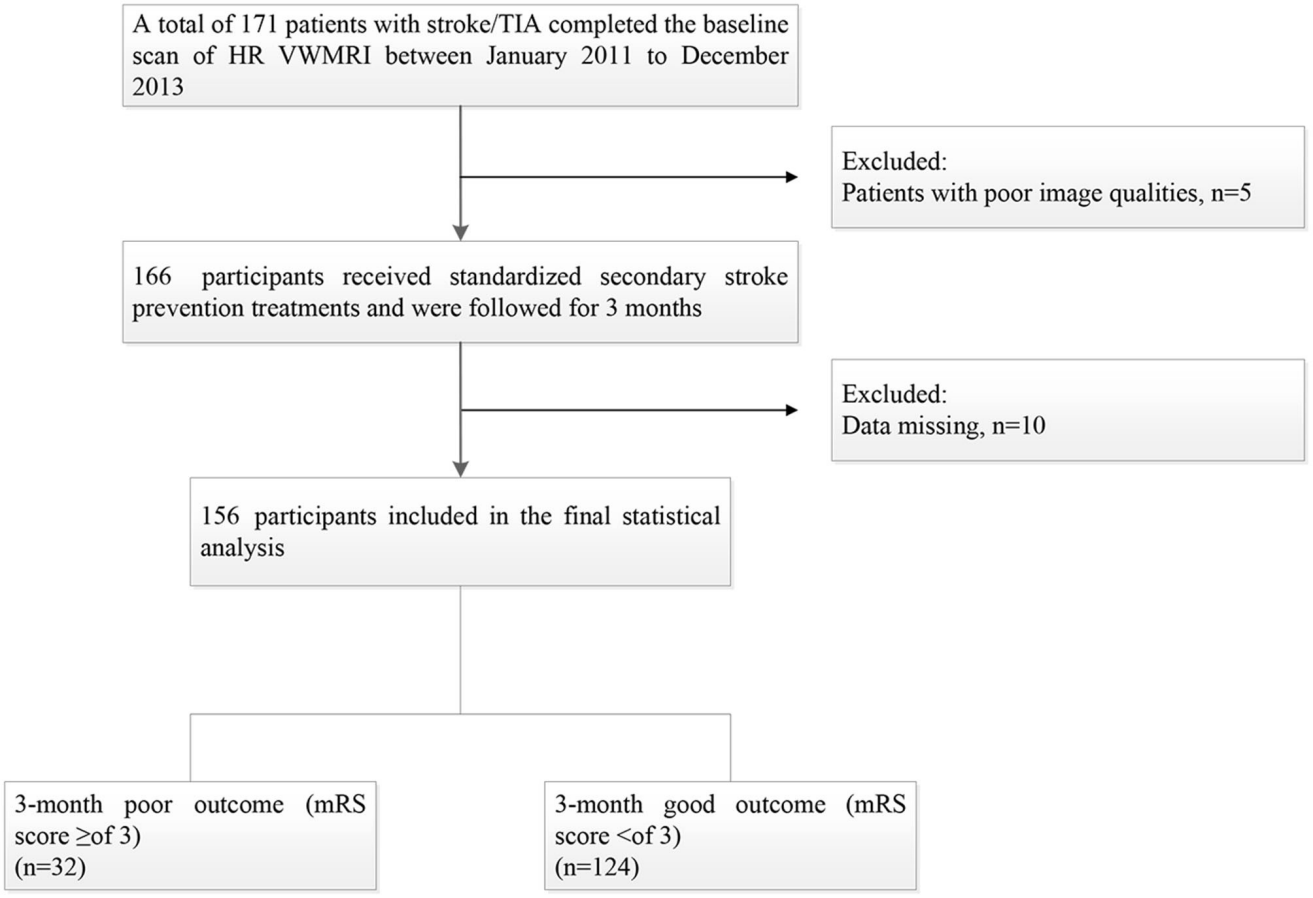
Flow chart of study patients.

One hundred seventy-one patients were enrolled from January 2011 to December 2013, and 15 patients were excluded for the following reasons: (1) 10 patients were lost; and (2) the image quality of 5 patients was poor. Thus, a total of 156 patients were included in the final statistical analysis, including 108 males (69.2%) with a mean age of (61.18 ± 10.12, 33–85) years. The median day of follow-up time was [IQR, 93 (78–110)]. Thirty-two patients (20.5%) had a 3-month poor prognosis (Figure 1).

**Figure 2:**
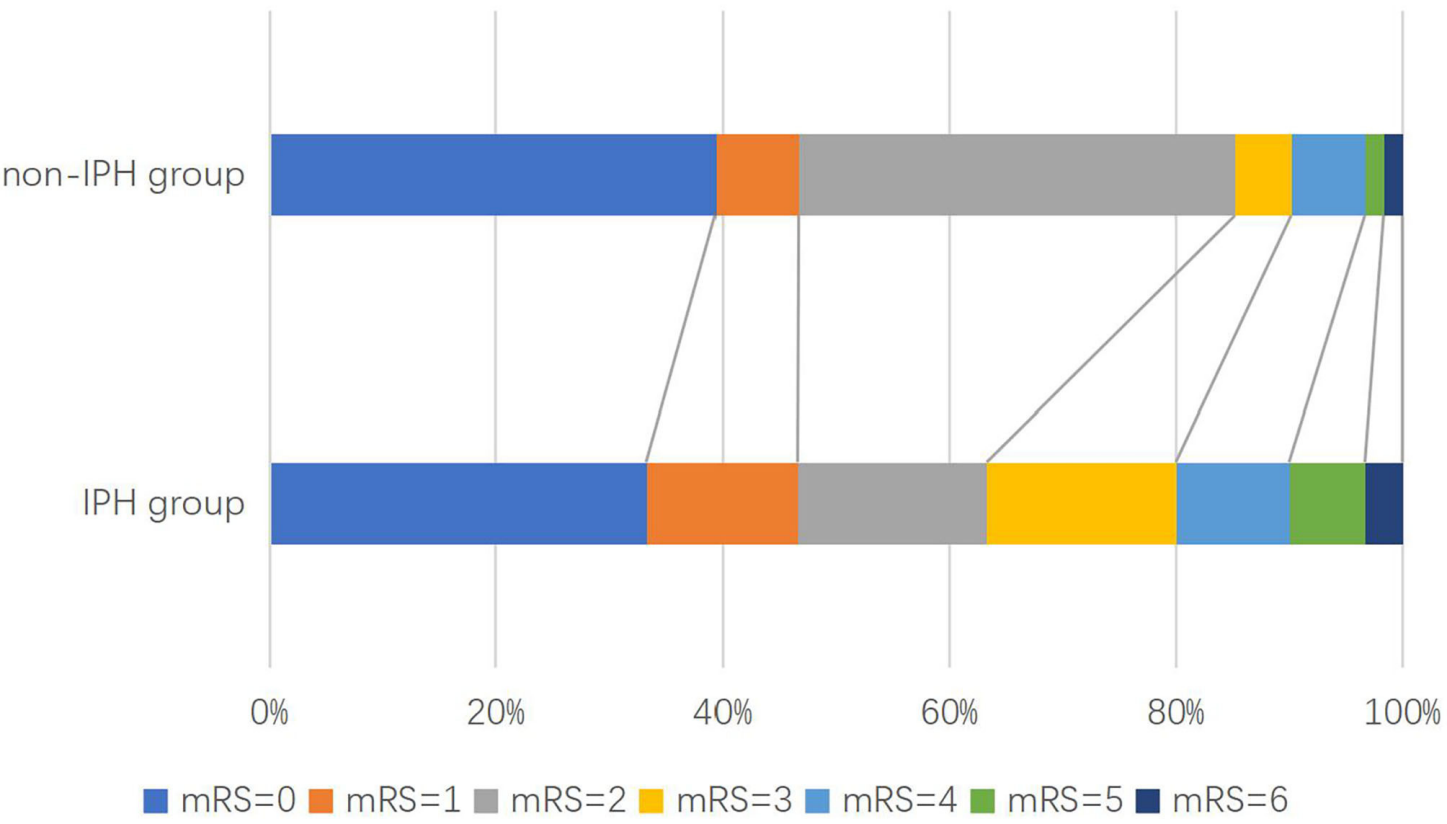
Distribution of modified Rankin scale (mRS) scores at 3 months in patients with acute ischemic stroke.

Intraplaque hemorrhage detected by HR VWMRI was found in 30 (20.5%) patients (Figure 2). There were significant differences in the age (mean, 66.30 ± 8.27 vs. 59.96 ± 10.16, *p* = 0.002), gender (male, 86.7 vs. 65.1%, *p* = 0.021), smoking history (60.0 vs. 31.7%, *p* = 0.004), NIHSS on admission [IQR, 3 (0–8) vs. 4 (2–12), *p* = 0.031], and DBP on admission (mean, 81.52 ± 11.40 vs. 86.94 ± 12.12 mmHg, *p* = 0.037) between the IPH group and the non-IPH group (Table 1).

**Table 1:** Demographic and clinical characteristics in patients.

**Variables**	**Total (*n* = 156)**	**IPH group (*n* = 30)**	**Non-IPH group (*n* = 126)**	***P-*value**
Gender, male, *n* (%)	108 (69.2)	26 (86.7)	82 (65.1)	**0.021**
Age, years	61.18 ± 10.12	66.30 ± 8.27	59.96 ± 10.16	**0.002**
Hypertension, *n* (%)	107 (68.6)	22 (73.7)	85 (67.5)	0.533
Diabetes mellitus, *n* (%)	50 (32.1)	13 (43.3)	37 (29.4)	0.141
Coronary heart disease, *n* (%)	18 (11.5)	2 (6.7)	16 (12.7)	0.528
Hyperlipidemia, *n* (%)	59 (37.8)	7 (23.3)	59 (37.8)	0.069
Prior stroke/TIA, *n* (%)	31 (19.9)	4 (13.3)	27 (21.4)	0.318
Smoking history, *n* (%)	58 (37.2)	18 (60.0)	40 (31.7)	**0.004**
Alcohol consuming, *n* (%)	51 (32.7)	9 (30.0)	42 (33.3)	0.726
Family history of stroke, *n* (%)	21 (13.5)	2 (6.7)	19 (15.1)	0.371
SBP on admission, mmHg	144.60 ± 21.75	143.15 ± 21.01	144.93 ± 21.99	0.702
DBP on admission, mmHg	85.92 ± 12.18	81.52 ± 11.40	86.94 ± 12.12	**0.037**
NIHSS on admission, IQR	4 (0–12)	3 (0–8)	4 (2–12)	**0.031**
Pre-admission mRS, IQR	1 (0–2)	1 (0–2)	1 (0–2)	0.059
hs-CRP, IQR, mg/L	2.60 (0.00–45.90)	3.30 (0.00–17.90)	2.50 (0.00–45.90)	0.617
TC, mmol/L	4.34 ± 1.07	4.25 ± 1.13	4.36 ± 1.06	0.634
TG, IQR, mmol/L	1.36 (0.50–8.44)	1.29 (0.67–3.89)	1.37 (0.50–8.44)	0.507
LDL-C, mmol/L	2.61 ± 0.96	2.39 ± 0.91	2.66 ± 0.96	0.184
HDL-C, mmol/L	1.07 ± 0.34	1.19 ± 0.56	1.05 ± 0.27	0.209
Hcy, IQR, umol/L	17.10 (6.60–75.00)	21.17 (10.50–51.20)	16.70 (6.60–75.00)	0.201
Stenosis rate of CA, %	62.77 ± 12.08	65.26 ± 14.32	62.18 ± 11.47	0.279
Stenotic degree of CA				**0.090**
Mild stenosis (<50%), *n* (%)	20 (12.8)	4 (13.3)	16 (12.7)	—
Moderate stenosis (50–69%), *n* (%)	92 (59.0)	13 (43.3)	79 (62.7)	—
Severe stenosis/occlusion (>70%), *n* (%)	44(28.2)	13 (43.3)	31 (24.6)	—
LA, mm^2^	16.86 ± 6.99	14.73 ± 7.94	17.37 ± 6.67	**0.063**
WA, IQR, mm^2^	30.65 (19.11–114.10)	34.56 (21.68–114.10)	29.61(19.11–54.03)	**<0.001**
VA, IQR, mm^2^	74.45 (43.00–162.11)	73.22 (43.00–162.11)	74.45(45.01–133.74)	0.269
NWI, IQR	0.42 (0.28–0.69)	0.46 (0.40–0.69)	0.41(0.28–0.63)	**<0.001**
WT, IQR, mm	1.11 (0.81–3.14)	1.33 (1.06–3.14)	1.07(0.81–1.80)	**<0.001**
PA, IQR, mm^2^	24.42 (8.60–74.29)	38.32 (11.42–71.35)	22.22 (8.60–74.29)	**<0.001**
RI	0.75 ± 0.08	0.68 ± 0.07	0.76 ± 0.07	**<0.001**
Calcification, *n* (%)	85 (54.5)	26 (86.7)	59 (46.8)	**<0.001**
Loose matrix, *n* (%)	74 (47.4)	24 (80.0)	50 (39.7)	**<0.001**
LRNC, *n* (%)	127 (81.4)	30 (100.0)	97 (77.7)	**0.004**
FCR, *n* (%)	4 (2.6)	4 (13.3)	0 (0.0)	**0.001**
Median on admission-to-following time, d (IQR)	93 (78–110)	93 (83–99)	93 (78–110)	0.750
mRS at 3 months, IQR	2 (0–5)	3 (0–5)	2 (0–5)	0.870
Medication at 3 months				
Aspirin, *n* (%)	89 (57.1)	19 (63.3)	70 (55.6)	0.439
Clopidogrel, *n* (%)	18 (11.5)	1 (3.3)	17 (13.5)	0.200
Statins, *n* (%)	68 (43.6)	8 (26.7)	60 (47.6)	0.038
Antihypertension, *n* (%)	49 (31.4)	9 (30.0)	40 (31.7)	0.853
Hypoglycemic, *n* (%)	47 (30.1)	11 (36.7)	36 (28.6)	0.385

*IPH, intraplaque hemorrhage; TIA, transient ischemic attack; SBP, systolic blood pressure; DBP, diastolic blood pressure; hs-CRP, hypersensitive c-reactive protein; TC, cholesterol; TG, triglycerides; LDL-C, low-density lipoprotein cholesterol; HDL-C, high-density lipoprotein cholesterol; Hcy, homocysteine; CA, carotid artery; LA, lumen area; WA, wall area; VA, vessel area; NWI, normalized wall index; WT, wall thickness; PA, plaque area; RI, remodeling index; LRNC, lipo-rich necrotic core; FCR, fibrous cap rupture; NIHSS, national institutes of health stroke scale; mRS, modified Rankin scale; IQR, interquartile range*.

Between the IPH group and the non-IPH group, there was no significant difference in the degree of stenosis (*p* = 0.279), lumen area (LA) (*p* = 0.063), and vessel area (VA) (*p* = 0.269). However, there were significant differences in the wall area (WA) [IQR, 34.56 (21.68–114.10) mm^2^ vs. 29.61 (19.11–54.03) mm^2^, *p* < 0.001], normalized wall index (NMI) [IQR, 0.46 (0.40–0.69) vs. 41 (0.28–0.63), *p* < 0.001], wall thickness (WI) [IQR, 1.33 (1.06–3.14) mm vs. 1.07 (0.81–1.80) mm, *p* < 0.001], plaque area (PA) [IQR, 38.32 (11.42–71.35) mm^2^ vs. 22.22 (8.60–74.29) mm^2^, *p* < 0.001], and remodeling index (RI) (mean, 0.68 ± 0.07 vs. 76 ± 0.07, *p* < 0.001) (Table 1).

**Figure 3:**
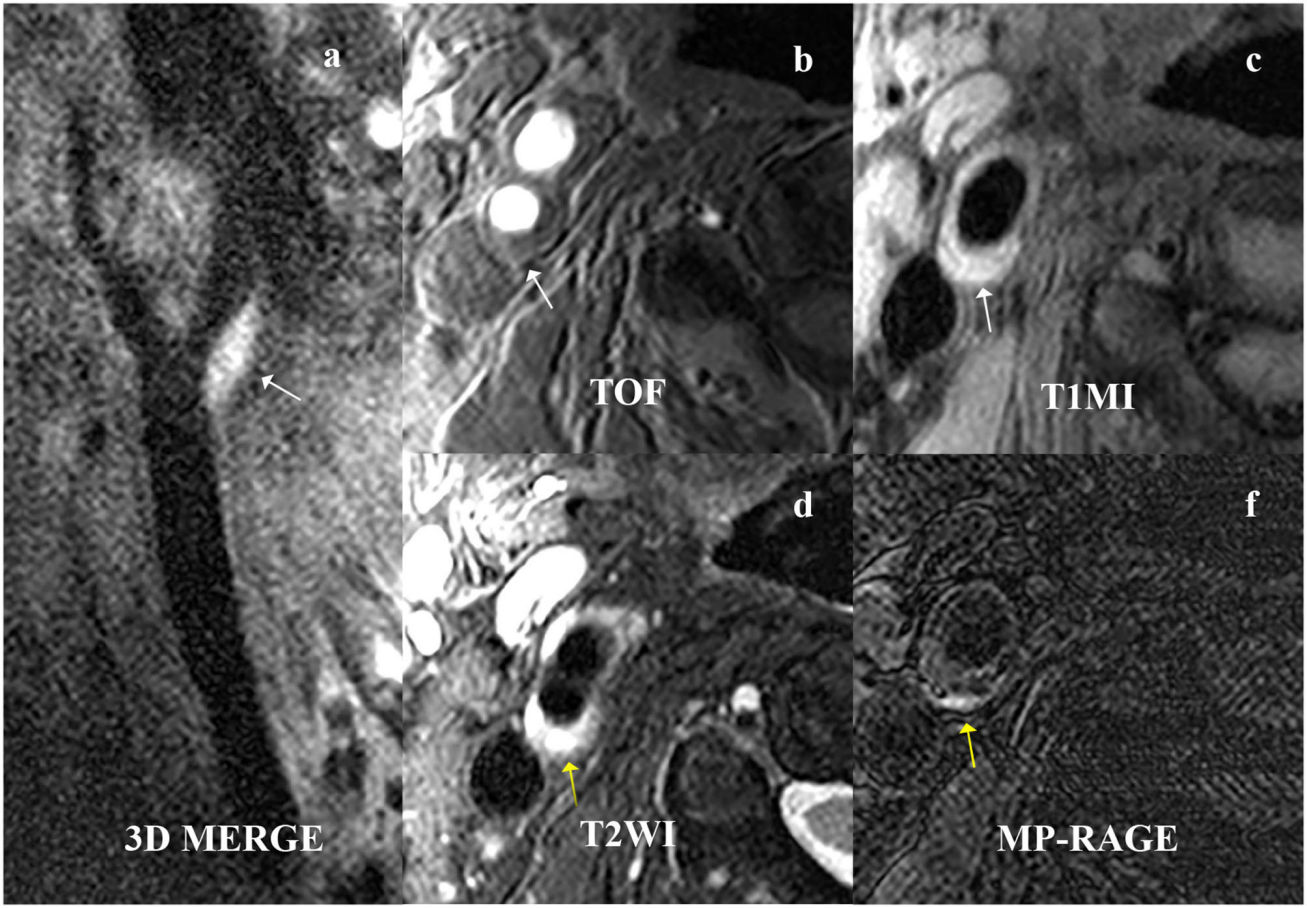
A 61-year-old male patient presented with right ischemic stroke. The plaque is shown at the beginning of the right internal carotid artery on **(a)** 3D MERGE, **(b)** TOF, and **(c)** T1W (white arrow). The intraplaque hemorrhage (IPH) is characterized by hyperintensities on **(d)** T2WI and **(e)** MPRAGE (yellow arrow). 3D MERG, 3D multiple-echo recalled gradient-echo; 3D-TOF, three-dimensional time of flight; T1W, T1-weighted; T2W, T2-weighted; MPRAGE, magnetization-prepared rapid acquisition gradient-echo.

During the follow-up, 32 patients (20.5%) had a poor functional outcome. The proportion of poor function outcome is 36.7% for the IPH group and 16.7% for the non-IPH group (crude OR 2.32; 95%CI 1.12–4.81; *p* = 0.024) (Figure 3). After adjusting the baseline variables as age, gender, smoking history, NIHSS on admission, DBP on admission, stenosis rate of CA, calcification, loose matrix, LRNC, and statins accepted at 3 months, there was still a statistical difference (adjusted OR 3.66; 95%CI 1.68–7.94; *p* = 0.001) between the two groups (Table 2). Regarding the second outcomes, the proportion of early stroke progression, early recurrent ischemic stroke, and hemorrhagic transformation in the IPH group was significantly higher (all *p* < 0.05, Table 2).

**Table 2:** Outcomes at 3 months after stroke onset in carotid plaque hemorrhage (IPH) group vs. non-IPH group.

**Outcome**	**No. (%) of patients**		**Unadjusted OR (95% CI)**	***P*-value**	Adjusted OR (95% CI)^*^	***P*-value**
	**IPH group (*n* = 30)**	**Non-IPH group (*n* = 126)**				
Primary outcome						
mRS 3–6 at 3 months	11 (36.7)	21 (16.7)	2.32 (1.12–4.81)	0.024	3.66 (1.68–7.94)	**0.001**
Second outcomes						
Early stroke progression	5 (16.7)	4 (3.2)	5.25 (1.41–19.60)	0.014	5.43 (1.45–20.25)	**0.012**
Early recurrent ischemic stroke	15 (50.0)	14 (11.1)	4.90 (2.37–10.17)	0.000	7.49 (3.13–17.94)	**<0.001**
Hemorrhagic transformation	1 (3.3)	3 (2.4)	1.51 (0.16–14.53)	0.722	-	**0.010**

* *Adjusted baseline variables: age, gender, smoking history, NIHSS on admission, DBP on admission, stenosis rate of CA, calcification, loose matrix, LRNC, and statins at 3 months*.

*mRS, modified Rankin Scale; IPH, intraplaque hemorrhage; CA, carotid artery; LRNC, lipo-rich necrotic core. OR, odds ratio; CI, confidence interval; mRS, modified Rankin Scale; IPH, intraplaque hemorrhage; CA, carotid artery; LRNC, lipo-rich necrotic core*.

## Discussion

Our study investigated the association between IPH detected by HR VWMRI and functional outcome at 3 months after stroke onset. Our study found that the incidence of carotid plaque IPH in patients with acute ischemic stroke was 19.27%. Meanwhile, the percentage of IPH was significantly higher than other plaque vulnerable features, which could reach 61.54%.

Our previous study has confirmed that IPH was associated with ipsilateral ischemic stroke recurrence, especially in the first 3 months after acute ischemic stroke. So, we hypothesized that IPH might be related to a 3-month poor prognosis. In our study, we found the proportion of patients with poor functional outcome in the IPH group is much higher than patients in the non-IPH group, which could be 3.66 times higher even after adjusting for confounding factors (including age, gender, smoking history, NIHSS on admission, DBP on admission, stenosis rate of CA, calcification, loose matrix, LRNC, and statins accepted at 3 months). However, we still wanted to know why the patients with IPH might be more prone to have a poor prognosis. So, we defined the early stroke progression, early recurrent ischemic stroke, and hemorrhagic transformation as the second outcomes. Our study found that patients with IPH might be more likely to occur early stroke progression, recurrent ischemic stroke, and hemorrhagic transformation.

Several studies have confirmed that IPH is associated with plaque progression (15, 16). Takaya et al. (17) followed the asymptomatic patients with carotid artery stenosis rate of 50–70% for 18 months. They found that the volume of the carotid artery wall and the percentage of lipid-rich necrotic core volume in the patients with IPH detected by MRI at baseline were significantly higher than in the control group. Meanwhile, the patients with IPH at baseline were also more likely to have new IPH at 18 months (43 vs. 0%; *p* = 0.006). According to the pathophysiological mechanism of plaque progression, some scholars believed that the hemoglobin would be released extracellularly after red blood cells are phagocytosed by macrophage. The free hemoglobin would increase inflammation, lipid core expansion, and oxidative stress, leading to plaque progression. IPH also carried proteolytic enzyme, leading to the degradation and destruction of the fibrous cap, and, finally, to the plaque rupture (18–20). Due to the limitations of this study, we did not study the pathophysiological process of IPH leading to the secondary outcomes, though it might be the research direction and hot spot in the future.

Our study still has some shortcomings. Firstly, because it is a single-center study with small sample size, the statistical results might be biased. Secondly, we did not make further study on the dynamic evolution of IPH, so we still do not know the pathophysiological mechanism of PH leading to poor functional outcome. Thirdly, our study did not exclude other risk factors affecting the prognosis of stroke, such as intracranial vascular artery disease, collateral circulation, and cerebral blood flow reserve capacity, which may be some errors in the results. Fourthly, we did not register the TOAST subtypes in our study, and it is a pity of the study. However, we have tried our best to rule out cardiogenic sources. We also hope to see the results of a large sample and more rigorous research in the future.

## Conclusion

Compared with patients without IPH, patients with IPH have a higher risk of poor 3-month outcome after stroke onset. Based on this, we should not overlook the potential risk of IPH. It might be essential to understand the underlying pathophysiological mechanism of plaque vulnerability and the molecular biomarkers combined with neuroimaging markers.

## Data Availability Statement

The original contributions presented in the study are included in the article/Supplementary Material, further inquiries can be directed to the corresponding author/s.

## Ethics Statement

The studies involving human participants were reviewed and approved by the Ethics Committee of Beijing Tiantan Hospital. Written informed consent to participate in this study was provided by the patient/participants or patient/participants' legal guardian/next of kin.

## Author Contributions

FC, XZ, XGo, BS, and YJ: concept and design. FC, YL, and XGe: drafting of the manuscript. XB and JJ: critical revision of the manuscript for important intellectual content. YL, BS, and YJ: provision of study material or patients. FC, YL, JJ, AW, XB, BS, YJ, and XGo: collection and assembly of data. XZ and BS: check and approve of clinical definition and supervision. FC and AW: data analysis. FC, AW, XB, BS, JJ, and XGe: data interpretation. XZ, BS, and YJ: administrative, and technical or material support. All authors: final approval of manuscript.

## Funding

The study was supported by grants from the Beijing Natural Science Foundation (Z200016).

## Conflict of Interest

The authors declare that the research was conducted in the absence of any commercial or financial relationships that could be construed as a potential conflict of interest.

## Publisher's Note

All claims expressed in this article are solely those of the authors and do not necessarily represent those of their affiliated organizations, or those of the publisher, the editors and the reviewers. Any product that may be evaluated in this article, or claim that may be made by its manufacturer, is not guaranteed or endorsed by the publisher.
