# Supplementary material for: Extracranial Carotid Plaque Hemorrhage Is Independently Associated With Poor 3-month Functional Outcome After Acute Ischemic Stroke—A Prospective Cohort Study
Source: Front Neurol. 2021 Dec 14;12:780436. doi: 10.3389/fneur.2021.780436 (PMC8712340; doi:10.3389/fneur.2021.780436)
Supplement: Supplementary file 2 [file Table_2.DOCX]

**Supplementary- Inclusion criteria and Exclusion criteria**

Inclusion criteria: (1) age ≥18 years old; (2) onset of symptoms within the last 14 days; (3) National Institutes of Health Stroke Scale score(NIHSS)≤22 when enrolled; (4) carotid intima-media-thickness (IMT) by ultrasound≥1.5mm. Exclusion criteria: (1) non-cerebral infarction disease including intracranial hemorrhage (intracranial parenchymal hemorrhage, subarachnoid hemorrhage, subdural hematoma or epidural hematoma), tumor, infection, etc.; (2) suspected cardiogenic embolism or previous history of congenital heart disease, rheumatic heart disease, atrial fibrillation, etc.; (3) suspected non-atherosclerotic vascular lesions ((i.e., vasculitis, arterial dissection, or Moyamoya disease); (4) undergoing emergent thrombolysis or interventional treatments; (5) previous history of carotid endarterectomy or carotid stenting; (6) suffering from severe hepatic or renal insufficiency; (7) life expectancy <1 year; (8) contraindication to MRI examination (including claustrophobia, metal placement in the body, etc.); (9) Severe stenosis or occlusion of the ipsilateral intracranial carotid artery or middle cerebral artery confirmed by magnetic resonance artery (MRA); (10) the image quality of HR VWMRI was too poor to be analyzed.
