# Supplementary material for: Extracranial Carotid Plaque Hemorrhage Is Independently Associated With Poor 3-month Functional Outcome After Acute Ischemic Stroke—A Prospective Cohort Study
Source: Front Neurol. 2021 Dec 14;12:780436. doi: 10.3389/fneur.2021.780436 (PMC8712340; doi:10.3389/fneur.2021.780436)
Supplement: Supplementary file 3 [file Table_3.DOCX]

**Supplementary-Clinical Information Assessment**

We obtained the pre-existing conditions of the patients from themselves, their relatives, or caregivers, and defined the present conditions following the definitions recommended by the related international guidelines: hypertension was defined by the diagnosis at discharge or a history of hypertension(23); diabetes mellitus was defined by the diagnosis at discharge or a history of diabetes mellitus(24); hyperlipidemia was defined as low-density lipoprotein (LDL) ≥ 1.7 mmol/L at admission or a history of hyperlipidemia or receiving lipid-lowering treatment or diagnosis at discharge(25); coronary artery disease was defined as the previous history of angina pectoris or myocardial infarct(26); smoking history was defined as continuous or cumulative smoking for more than six months in one's life(27); family history of stroke was defined as at least one of a patient's first-grade relatives having a history of stroke(28).
