# Supplementary material for: Extracranial Carotid Plaque Hemorrhage Is Independently Associated With Poor 3-month Functional Outcome After Acute Ischemic Stroke—A Prospective Cohort Study
Source: Front Neurol. 2021 Dec 14;12:780436. doi: 10.3389/fneur.2021.780436 (PMC8712340; doi:10.3389/fneur.2021.780436)
Supplement: Supplementary file 4 [file Table_4.DOCX]

**Carotid MR Imaging Protocol**

All patients underwent a routine brain MRI scan and carotid ultrasonographic screen to define the symptomatic carotid artery. Then, they were performed by carotid MR imaging on a 3T MR scanner (Achieva TX; Philips Healthcare, Best, the Netherlands) with 8-channel head coils. A standardized multisequence protocol was performed for the carotid artery bifurcation on the symptomatic side by acquiring three-dimensional time of flight (3D-TOF), T1-weighted (T1W), T2-weighted (T2W), and magnetization-prepared rapid acquisition gradient echo (MPRAGE) sequences. The contrast material was gadolinium diethylenetriamine pentametric acid (GD-DTPA) at an injection dose of 0.1mmol/Kg. In addition, delayed T1WI scanning was performed for about 5 minutes after intravenous injection. Finally, spectral preservation attenuated inversion recovery (SPAIR) was used in the black blood technique to enhance the contrast between the vessel wall and the surrounding tissue. The minimum resolution of each sequence plane reconstruction was 0.55mm × 0.55mm. Therefore, the total scanning time was about 35 minutes. The imaging parameters are as follows: (1) 3D-TOF MRA: repetition Time (TR)/echo Time (TE)=20/4.9ms, Flip Angle (FA)=20; Field of view (FOV)=140×140mm, matrix size=256×256, slice thickness=1.0mm; (2) T1W: TR/TE=800/10ms, FA=90, FOV=140mm×140mm, Matrix size=256×256, slice thickness=2.0mm; (3) T2W: TR/TE=4800/50ms, FA=90; FOV=140mm×140mm, matrix size=256×256, slice thickness=2.0mm; (4)MP-RAGE: TR/TE=8.8/5.3ms, FA=15, FOV=140mm×140mm, Matrix size=256×256, slice thickness=1.0mm
