# Supplementary material for: Extracranial Carotid Plaque Hemorrhage Is Independently Associated With Poor 3-month Functional Outcome After Acute Ischemic Stroke—A Prospective Cohort Study
Source: Front Neurol. 2021 Dec 14;12:780436. doi: 10.3389/fneur.2021.780436 (PMC8712340; doi:10.3389/fneur.2021.780436)
Supplement: Supplementary file 5 [file Table_5.DOCX]

**MRI Image Analysis**

We used a standard workstation (ADW4.4, G.E. Medical System, USA) for imaging analysis. We defined the plaque as a thickening of the focal wall relative to image slices from beneath the T2- and T1-weighted imaging focal wall. First, we manually outlined the vessel and lumen. Then, we measured the parameters of plaque burden and the stenosis ratio of the carotid. We detected the plaque components (including calcification, lipo-rich necrotic core, IPH, and fibrous cap rupture) and quantitatively analyzed them. According to the international standards of MRI (29), calcification is characterized by hypointensities on TOF, T1W, T2W, and MPRAGE sequences; lipid-rich necrotic core (LRNC) manifests isointensities on TOF, T1W and T2W, and hypointensity on MPRAGE; IPH manifests hyperintensities on TOF, T1W and MPRAGE, and isointensity to hyperintensity on T2W. The image quality was divided into four grades: grade 1=the image of the carotid lumen and the outside of the blood vessel was not clear, or the signal-noise ratio (SNR) was low, and there were noticeable motion artifacts; Grade 2=the carotid artery wall was visible in the image, but the wall structure and the external contour of the vessel could not be judged, and SNR was low, or there were motion artifacts; Grade 3=the carotid artery wall structure, lumen, and outside vascular contour were clear, the blood flow signal in the lumen was not wholly suppressed, and SNR was high, accompanied by a small amount of movement or swallowing artifacts; Grade 4=the carotid artery wall structure, lumen, and outside vascular contour were clear, the blood flow signal in the lumen was wholly suppressed, and SNR was high without apparent artifacts. Therefore, the image quality of≤grade 2 was excluded from the statistical analysis. Two experienced senior neuroimaging physicians reviewed all slices of the sequences of carotid plaques on HR VWMRI, blinded to the clinical information and the diffusion-weighted images (DWI). Consensus interpretation was accepted for the final analysis if the two readers' interpretations were different. According to the reproducibility test, we randomly selected twenty patients from the study population every two months to test the consistency of inter-reader and intrareader in measuring carotid plaque morphology and carotid plaque compositions.
