# Supplementary material for: Extracranial Carotid Plaque Hemorrhage Is Independently Associated With Poor 3-month Functional Outcome After Acute Ischemic Stroke—A Prospective Cohort Study
Source: Front Neurol. 2021 Dec 14;12:780436. doi: 10.3389/fneur.2021.780436 (PMC8712340; doi:10.3389/fneur.2021.780436)
Supplement: Supplementary file 6 [file Table_6.DOCX]

**Consistency of Inter-reader and Intrareader**

The consistency of the inter-reader for two measurement results was well. (VA in reference slice (ICC=0.896; 95％CI, 0.67-0.99), LA in reference slice(ICC=0.845; 95％CI, 0.54-0.96), VA in the narrowest slice(ICC=0.902; 95％CI, 0.71-0.98), LA in the narrowest slice(ICC=0.883; 95％CI, 0.57-0.98).The consistency of the intrareader for two measurement results was well, too. (VA in reference slice(ICC=0.893; 95％CI, 0.67-0.98), LA in reference slice(ICC=0.802; 95％CI, 0.42-0.97), VA in the narrowest slice(ICC=0.836; 95％CI, 0.62-0.996), LA in the narrowest slice(ICC=0.812; 95％CI, 0.56-0.94).
